# Viral concentration method biases in the detection of viral profiles in wastewater

**DOI:** 10.1128/aem.01339-24

**Published:** 2024-12-06

**Authors:** Naeema Cheshomi, Absar Alum, Matthew F. Smith, Efrem S. Lim, Otakuye Conroy-Ben, Morteza Abbaszadegan

**Affiliations:** 1 School of Sustainable Engineering and the Built Environment, Arizona State University 310018 , Tempe, Arizona, USA; 2 Water and Environmental Technology Center, Arizona State University 7864 , Tempe, Arizona, USA; 3 Center for Fundamental and Applied Microbiomics, Biodesign Institute, Arizona State University 43363 , Tempe, Arizona, USA; Centers for Disease Control and Prevention, Atlanta, Georgia, USA

**Keywords:** wastewater-based epidemiology, viral concentration method, enveloped/non-enveloped virus, next-generation sequencing, wastewater sample matrix

## Abstract

Viral detection methodologies used for wastewater-based epidemiology (WBE) studies have a broad range of efficacies. The complex matrix and low viral particle load in wastewater emphasize the importance of the concentration method. This study focused on comparing three commonly used virus concentration methods: polyethylene glycol precipitation (PEG), immuno-magnetic nanoparticles (IMNP), and electronegative membrane filtration (EMF). Influent and effluent wastewater samples were processed by the methods and analyzed by DNA/RNA quantification and sequencing for the detection of human viruses. SARS-COV-2, Astrovirus, and Hepatitis C virus were detected by all the methods in both sample types. PEG precipitation resulted in the detection of 20 types of viruses in influent and 16 types in effluent samples. The corresponding number of virus types detected was 21 and 11 for IMNP, and 16 and 8 for EMF. Certain viruses were unique to only one concentration method. For example, PEG detected three types of viruses in influent and six types in effluent compared to IMNP, which detected seven types in influent and one type in effluent samples. However, the EMF method appeared to be the least effective, detecting three types in influent and none in effluent samples. Rotavirus was detected in influent sample using IMNP method, whereas EMF and PEG methods failed to yield a similar outcome. Consequently, the potential false negative results pose a risk to the credibility of WBE applications. Therefore, implementation of a proper concentration technique is critical to minimize method biases and ensure accurate viral profiling in WBE studies.

**IMPORTANCE:**

In recent years, significant research efforts have been focused on the development of viral detection methodology for wastewater-based epidemiology studies, showing a range of variability in detection efficacies. A proper methodology is essential for an appropriate evaluation of disease prevalence and community health in such studies and necessitates designing a concentration method based on the target pathogenic virus. There remains a need for comparative performance evaluations of methods in the context of detection efficiencies. This study highlights the significant impact of sample matrix, viral structure, and nucleic acid composition on the efficacy of viral concentration methods. Assessing WBE techniques to ensure accurate detection and understanding of viral presence within wastewater samples is critical for revealing viral profiles in municipality wastewater samples.

## INTRODUCTION

Wastewater-based epidemiology (WBE) is a promising tool that provides access to valuable information about population-level epidemiology. WBE helps elucidate the health condition of a community, enhances our understanding of disease prevalence and circulation, provides early warning of epidemics, and discloses credible information to public health authorities (1–4). Moreover, WBE has the potential to complement clinical surveillance by estimating and reflecting the actual disease prevalence (1, 5), even for conditions that may be missed by clinical systems due to limited healthcare engagement, testing capacities, or even dormant symptoms in some viral diseases, such as HIV, HPV, etc. For example, research conducted by Pillay et al. (6) estimated that a higher number of COVID-19-infected people was expected, compared with reported clinical testing data. In addition, current methods applied to community wastewater at municipal wastewater treatment plant (WWTP) do not violate privacy rights.

Wastewater samples have historically been used to detect the presence and outbreak of human viral pathogens, such as poliovirus, norovirus, enterovirus, rotavirus, adenovirus, and hepatitis A and E (3, 7, 8). More recently, wastewater-based environmental surveillance has been successfully used for large-scale investigation of the presence and prevalence of the variants of SARS-CoV-2, particularly during the COVID-19 pandemic (9).

Wastewater is a challenging medium for the detection of viral pathogens, especially due to a high level of assay inhibitors for both culture-based and molecular methods. Selecting an appropriate viral detection methodology, including concentration and end point detection, is essential to properly detect target viruses in a low load and a complex wastewater sample matrix (7). Various methods have been studied for the concentration and purification of different types of enteric viruses and viral surrogates in wastewater samples (10–12), including electrostatically charged membrane filtration (4), ultrafiltration (13), polyethylene glycol (PEG) precipitation (14), aluminum-based adsorption-precipitation (7), flocculation (15), and ultracentrifugation (12, 16).

Guzmán et al. (11) showed the impact of concentration method on the overall efficacy of viral detection using an aluminum-based procedure and the commercially available Maxwell RSC Enviro Wastewater TNA Kit (TNA) (7). As demonstrated by this study and others, the credibility of WBE techniques is reliant on the accuracy and sensitivity of the methods used, which is limited by the disparity and variability of the data reported in different WBE studies (17, 18). Therefore, there remains a need for a comparative evaluation of methods used in WBE studies, considering the factors playing a critical role in the performance of viral detection in wastewater samples. This study investigates the efficacy of concentration methods as impacted by viral diversity, including structure, nucleic acid composition, and low particle load. Next-generation sequencing was used to analyze the presence of 106 various types of viruses (201 different strains). The detection panel includes 40 respiratory viruses (Influenza, Rhinovirus, Human coronaviruses, etc) and 66 viruses of different public health importance (GI pathogens: norovirus, rotavirus, polio, etc; oncogenic viruses: HPV, polyomaviruses, etc; mosquito-borne viruses: west nile, zika, dengue, etc; and respiratory viruses: Influenza, Rhinovirus, Human coronaviruses, etc).

## MATERIALS AND METHODS

### Wastewater sample collection

Wastewater samples were collected from a municipal wastewater treatment facility in central Arizona during the month of May 2023. The facility has a treatment capacity of 18 million gallons per day (MGD) and treats wastewater to state and federal standards through physical and biological processes. Specifically, wastewater is screened to remove inorganic debris, followed by primary clarification to remove organic solids, and processed through an aerobic biological process, which reduces total nitrogen to under 10 mg.L^−1^. It is additionally treated by secondary clarification and filtration through 10 μm cloth media and finally disinfected by ultraviolet (UV). Two liters of the influent (untreated) and the clarified tertiary-treated effluent (disinfected/post-UV) samples were collected in sterilized polypropylene bottles (cat.no. 112 0500BPC, Thermo Fisher Scientific). The samples were transported to the lab at 4°C on ice and kept at 4°C for 24 h until processed.

### Sample processing

Three virus concentration methods, immunomagnetic nanoparticles (IMNP), electronegative membrane filtration (EMF) (19), and polyethylene glycol precipitation (PEG) (20), were employed to concentrate viruses in both influent and effluent wastewater samples as described below. For this comparative study, two types of samples (influent and effluent) were processed in parallel by three concentration methods. Three aliquots of each sample were processed in triplicate by repeating nucleic acid extraction, concentration method, PCR amplification, and subsequent sequencing. Since the focus of this study was to compare the efficacy of virus concentration methods, influent and effluent samples were included for representation of the virome of raw and treated wastewater. The concentration methods and virus types were the principal variables, whereas the detection/quantification methods (PCR and sequencing) were the secondary variables.

#### PEG method

Wastewater samples (200 mL) were shaken for 2–3 min and allowed to settle for 45 min at room temperature to precipitate large-sized particles. The supernatant (150 mL) was collected, and 50 mL aliquots (triplicate) were transferred in a low-density polypropylene bottle (cat.no. 112–0120, Thermo Fisher Scientific). Molecular biology-grade PEG 8000 (10% wt/vol; cat.no. BP-100, Fisher Scientific) and 1.0 M NaCl (wt/vol) (21) (cat.no. S7653-1KG, Sigma-113 Aldrich) were added to the samples. The bottles were gently mixed overnight (12 h) at 4°C on a stir plate (cat.no. SH4000-S, Southwest Science). The bottles were centrifuged (Sorvall RC5CPlus) in swing buck rotor (cat.no. PN11788, Sorvall) at 4500 rpm (4330 g force) for 30 min at 4°C to pellet the samples. The supernatant was discarded, and the pellets were carefully collected and resuspended in AVL buffer, which is provided by the QIAamp Viral RNA Mini kit (cat.no. 52906, Qiagen, San Diego, CA, USA), and then promptly processed for RNA extraction.

#### EMF method

Fifty-milliliter aliquots (triplicate) of the clarified influent and effluent wastewater samples were supplemented with MgCl_2_ (cat.no. M8266-100G, Sigma) to achieve a final concentration of 25 mM. The resulting mixture was then filtered through a 0.45-µm-pore-size, 47-mm-diameter electronegative mixed cellulose-ester membrane (cat.no. AAWP09000, Merck Millipore, Burlington, MA, USA). Thereafter, the filter was carefully rolled, placed in a 25 mL conical tube, and stored at – 20°C for subsequent nucleic acid extraction.

#### IMNP method

Wastewater samples (200 mL) were shaken for 2–3 min and allowed to settle for 45 min at room temperature to precipitate large-sized particles. Three 50 mL aliquots of each clarified influent and effluent sample were processed by 750 µL of Nanotrap Microbiome Particles (cat.no. 44202, Ceres Nanosciences), and the samples were inverted (2 times) for thorough mixing. The samples were then incubated at room temperature for 30 min and inverted every 5 min, and then placed in a DynaMag-50 magnetic rack (cat.no. 12302D, Thermo Fisher Scientific) for 10 min to separate the magnetic nanoparticles. The supernatant was carefully discarded, and the pellet was resuspended in molecular grade water (cat.no. MT46000CI, Corning) and transferred to a 2 mL microcentrifuge tube. The microcentrifuge tube was placed on a DynaMag-2 magnetic rack (cat.no. 12321D, Thermo Fisher Scientific) for 2 min to separate the Nanotrap particles from the sample. After adding the buffer to the Nanotrap particle pellet, the sample was heated, and the 2 mL centrifuge tube was placed in a DynaMag-50 magnetic rack to isolate the supernatant. The supernatant was then collected in a new 2 mL collection tube and promptly processed for nucleic acid extraction, as described below.

### Viral nucleic acid extraction and quantification

The wastewater samples concentrated by PEG and IMNP methods were processed for nucleic acid extraction using the QIAamp Viral RNA Mini Kit (cat.no. 52906, Qiagen, San Diego, CA, USA). The wastewater samples concentrated by the EMF methods were processed for nucleic acid extraction using the RNeasy PowerWater Kit (cat.no. 14700–50-NF, Qiagen, San Diego, CA, USA). Both QIAamp and RNeasy methods were modified to extract both DNA and RNA viral genomes simultaneously by eliminating the DNase I Solution addition. All extracted nucleic acids were stored at –80°C for downstream applications.

### Next-generation sequencing and bioinformatics

Next-generation sequencing libraries were constructed from wastewater-derived nucleic acids using the RNA Prep with Enrichment kit (Illumina, San Diego, CA, USA) and hybridization-capture-based virus enrichment with the Illumina Viral Surveillance Panel, according to the manufacturer-provided protocol. Libraries were quantified using the fluorometric Qubit 1X dsDNA assay kit and Qubit Flex instrument (Invitrogen, Waltham, MA, USA) and sequenced on an Illumina NextSeq 2000 instrument at 151 cycles of paired-end reads.

Demultiplexing of fastq files was performed with Illumina DRAGEN software BCL Convert (version 3.8.4). Fastq quality control workflow was performed with the BBTools suite (22). Briefly, adapter trimming, read quality and length filtering, and contaminant removal (PhiX) were performed using BBDuk specifying a minimum length of 75 bases and a minimum Phred Quality score of 20. Reads with at least 99% identity to another read were filtered out using Dedupe. Overlapping reads were joined using BBMerge before a second round of deduplication with Dedupe on merged reads specifying a minimum identity of 100% to demark duplicates. All remaining reads (deduplicated merged and unmerged read pairs) were passed through BBDuk a final time to remove BBDuk-default Illumina sequences. Quality-filtered fastq files were converted to fasta format, and the reads were mapped to a custom database containing representative reference sequences for viruses included in the enrichment panel using Bowtie 2 (23) to generate SAM format alignments. Non-primary, supplementary, and unmapped reads were filtered from the primary alignment using SAMtools view (24). Primary SAM format alignments were converted to BAM format and indexed using SAMtools sort and index. Mapping statistics were computed from each primary BAM file using SAMtools idxstats. Calculation of normalized read counts (Reads Per Million; RPM) for all samples was performed in R statistical software (25). Three aliquots of each concentrated sample were processed in triplicate for nucleic acids extraction, followed by PCR amplification and subsequent sequencing.

## RESULTS AND DISCUSSION

The comparative performance of the three methods for capture and recovery of viruses is presented based on abundance and variety of different viruses in influent and effluent wastewater samples collected from a local wastewater treatment facility.

### Variety and abundance of the detected viruses

The data on PCR and Illumina NextSeq 2000 sequence analyses of influent and effluent samples processed in parallel using three virus concentration methods are presented in this section. A total of 35 viruses were identified in the wastewater influent and effluent. The detected viruses included both the enveloped and non-enveloped viruses belonging to the following families: *Flaviviridae, Picornaviridae, Reoviridae, Caliciviridae, Coronaviridae, Astroviridae, Poxviridae, Adenoviridae, Anelloviridae, Polyomaviridae, Papillomaviridae,* and *Sedoreoviridae*.

**Fig 1:**
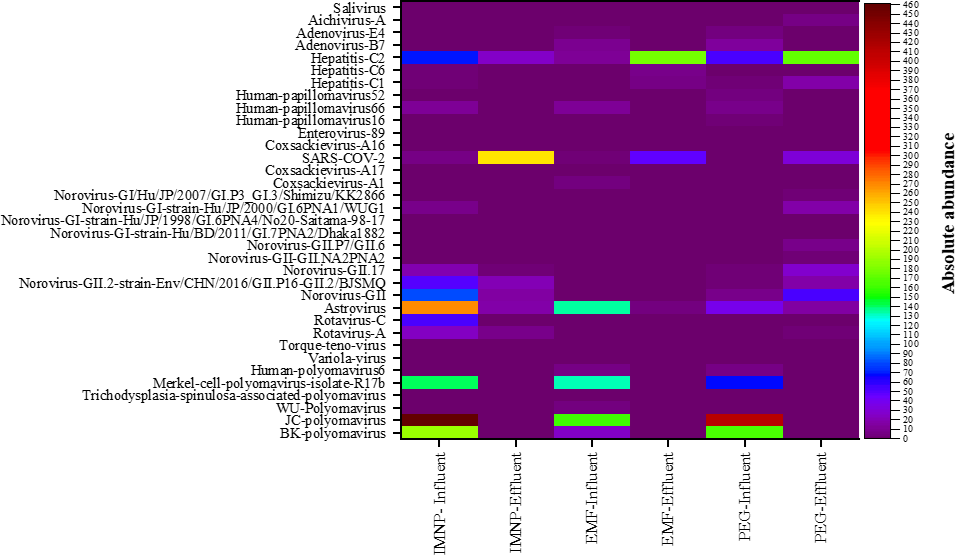
Heatmap of viral composition distributed in the influent and the effluent samples.

The impact of the viral concentration method on the diversity and abundance of viruses and the corresponding genome reads is summarized in the heatmap plot (Fig. 1). The results indicate dissimilarities between the types of viruses identified in the influent and effluent samples. By all three methods, JC polyomavirus was only detected in the influent samples, whereas SARS-COV-2 was detected with higher abundance in the effluent compared with the influent samples.

**Fig 2:**
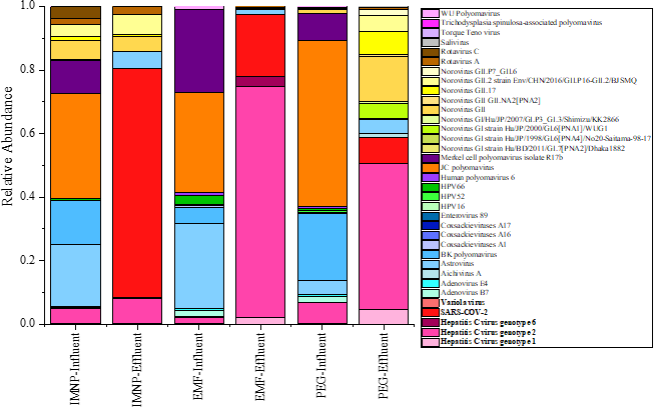
Relative abundance of viral communities in the influent and the effluent samples.

The relative abundance of viruses in both influent and effluent samples elucidates the relative efficacy of the three concentration techniques over the broad spectrum of viral profiles (Fig. 2). JC polyomavirus had the highest relative abundance in the influent samples processed by all three methods. Conversely, in effluent samples, Hepatitis C had the highest relative abundance in samples processed by EMF and PEG methods, whereas SARS-COV-2 had the highest relative abundance in effluent samples concentrated by the IMNP method (26). Our results illustrate that different viral concentration methods produce distinct viral profiles, highlighting the significance of enrichment methodology in viral detection in wastewater (Fig. 2). These results are consistent with the previous studies reporting that the detection methods, for instance, using different DNA library preparation kits, can result in significant differences in ratios and types of viral families detected in wastewater (26).

**Fig 3:**
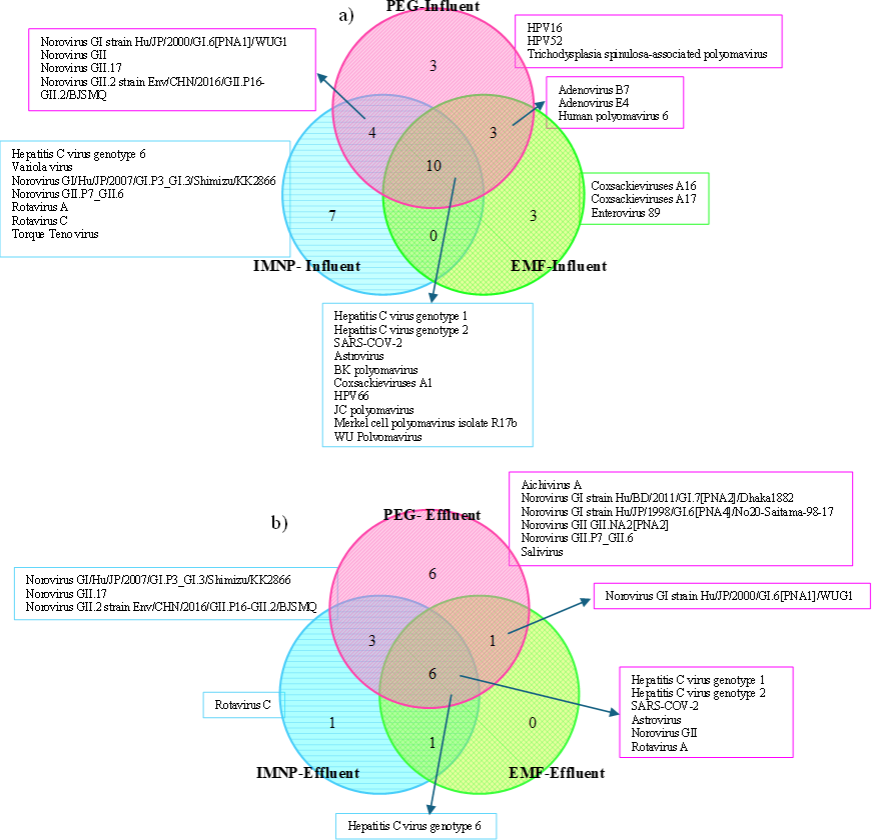
Venn diagram of viral distribution in (a) influent and (b) effluent samples.

The similarities and differences in the distribution of detected viruses using each concentration method in both influent and effluent samples are represented in Venn diagrams (Fig. 3a and b). Interestingly, variations in virus distribution based on the applied method are observable even when processing unique samples from each point. In Fig. 3a, 10 viruses were common among all methods (Hepatitis C virus genotype 1, Hepatitis C virus genotype 2, SARS-COV-2, Astrovirus, BK polyomavirus, Coxsackieviruses A1, HPV66, JC polyomavirus, Merkel cell polyomavirus isolate R17b, and WU Polyomavirus). Three viruses were exclusively detected by PEG and IMNP methods (variants of Norovirus, including Norovirus GI strain Hu/JP/2000 /GI.6[PNA1]/WUG1, Norovirus GII, Norovirus GII.17, and Norovirus GII.2 strain Env/CHN/2016/GII.P16-GII.2/BJSMQ). Adenovirus B7, Adenovirus E4, and Human polyomavirus 6 were identified exclusively by PEG and EMF methods, with no detection in IMNP-processed samples.

Furthermore, the results reveal that certain viruses were unique to only one concentration method. Notably, Hepatitis C virus genotype 6, Variola virus, Norovirus GI/Hu/JP/2007 /GI.P3_GI.3/Shimizu/KK2866, Norovirus GII.P7_GII.6, Rotavirus A, Rotavirus C, and Torque Teno virus were only identified in the influent samples when utilizing the IMNP method. Similarly, for HPV16, HPV52, and Trichodysplasia spinulosa-associated polyomavirus were detected when utilizing the PEG method, and Coxsackieviruses A16, Coxsackieviruses A17, and Enterovirus 89 were exclusively detected in samples processed using the EMF method. These findings emphasize that selecting the appropriate viral concentration method should be on the target virus type, particularly in influent samples where various components coexist. In essence, the lack of viral genome in the sequencing data does not necessarily indicate the absence of the virus in the sample, underlining the critical importance of using an appropriate viral concentration method tailored to the target virus.

The results reveal that 18 distinct viruses were identified in effluent wastewater samples (Fig. 3b), whereas 30 viruses were detected in influent sample, with five of these viruses exclusively detected in effluent samples. Six different viruses, namely Hepatitis C virus genotype 1, Hepatitis C virus genotype 2, SARS-COV-2, Astrovirus, Norovirus GII, and Rotavirus A, were detected in samples processed by PEG, IMNP, and EMF methods. Norovirus GI/Hu/JP/2007 /GI.P3_GI.3/Shimizu/KK2866, Norovirus GII.17, and Norovirus GII.2 strain Env/CHN/2016/GII.P16-GII.2/BJSMQ were detected by both PEG and IMNP methods. Norovirus GI strain Hu/JP/2000 /GI.6[PNA1]/WUG1 was a commonly detected virus in samples processed with PEG and EMF methods. Both IMNP and EMF methods detected hepatitis C virus genotype 6.

In wastewater effluent, Aichivirus A, Norovirus GI strain (Bangladesh isolate), Norovirus GI strain (Japan Isolate), Norovirus GII GII.NA2[PNA2], Norovirus GII.P7_GII.6, and Salivirus were detected only by the PEG isolation. Moreover, only the IMNP method was selected for Rotavirus C in effluent wastewater. Based on these findings for the effluent samples, we can conclude that the EMF method is not recommended as it did not detect any unique viruses exclusively. In contrast, the other methods efficiently identified all detected viruses in the effluent sample and demonstrated effectiveness across a broader range of viruses, and there is no one-size-fits-all concentration method. Therefore, the choice of concentration method should be tailored to the specific target virus. The virion-specific information and classification of the detected viruses across all samples are summarized in Table 1.

**TABLE 1:** Classification of the detected viruses in the influent and the effluent samples

Detected viruses	Structure			Detected in wastewater					
	Enveloped	Non-enveloped	Genome structure	Influent			Effluent		
				IMNP	EMF	PEG	IMNP	EMF	PEG
Hepatitis C virus genotype 1	●		ssRNA	●	●	●	●	●	●
Hepatitis C virus genotype 2	●		ssRNA	●	●	●	●	●	●
Hepatitis C virus genotype 6	●		ssRNA	●	−	−	●	●	−
SARS-COV-2	●		ssRNA	●	●	●	●	●	●
Variola virus	●		dsDNA	●	−	−	−	−	−
Adenovirus B7		●	dsDNA	−	●	●	−	−	−
Adenovirus E4		●	dsDNA	−	●	●	−	−	−
Aichivirus A		●	ssRNA	−	−	−	−	−	●
Astrovirus		●	ssRNA	●	●	●	●	●	●
BK polyomavirus		●	dsDNA	●	●	●	−	−	−
Coxsackieviruses A1		●	ssRNA	●	●	●	−	−	−
Coxsackieviruses A16		●	ssRNA	−	●	−	−	−	−
Coxsackieviruses A17		●	ssRNA	−	●	−	−	−	−
Enterovirus 89		●	ssRNA	−	●	−	−	−	−
HPV16		●	dsDNA	−	−	●	−	−	−
HPV52		●	dsDNA	−	−	●	−	−	−
HPV66		●	dsDNA	●	●	●	−	−	−
Human polyomavirus 6		●	ssDNA	−	●	●	−	−	−
JC polyomavirus		●	dsDNA	●	●	●	−	−	−
Merkel cell polyomavirus isolate R17b		●	dsDNA	●	●	●	−	−	−
Norovirus GI strain Hu/BD/2011 /GI.7[PNA2]/Dhaka1882		●	ssRNA	−	−	−	−	−	●
Norovirus GI strain Hu/JP/1998 /GI.6[PNA4]/No20-Saitama-98–17		●	ssRNA	−	−	−	−	−	●
Norovirus GI strain Hu/JP/2000 /GI.6[PNA1]/WUG1		●	ssRNA	●	−	●	−	●	●
Norovirus GI/Hu/JP/2007 /GI.P3_GI.3/Shimizu/KK2866		●	ssRNA	●	−	−	●	−	●
Norovirus GII		●	ssRNA	●	−	●	●	●	●
Norovirus GII GII.NA2[PNA2]		●	ssRNA	−	−	−	−	−	●
Norovirus GII.17		●	ssRNA	●	−	●	●	−	●
Norovirus GII.2 strain Env/CHN/2016/GII.P16-GII.2/BJSMQ		●	ssRNA	●	−	●	●	−	●
Norovirus GII.P7_GII.6		●	ssRNA	●	−	−	−	−	●
Rotavirus A		●	dsRNA	●	−	−	●	●	●
Rotavirus C		●	dsRNA	●	−	−	●	−	−
Salivirus		●	ssRNA	−	−	−	−	−	●
Torque Teno virus		●	ssDNA	●	−	−	−	−	−
Trichodysplasia spinulosa-associated polyomavirus		●	dsDNA	−	−	●	−	−	−
WU polyomavirus		●	dsDNA	●	●	●	−	−	−

### Factors affecting the efficacy of viral concentration methods

Identifying the key factors that may influence viral concentration efficiency is critical in selecting the most suitable viral concentration method and avoiding any potential false negative results (27). Several characteristics can impact the efficiency of applied concentration and detection methods. These include the structure of nucleic acids and the presence of an additional lipid bilayer membrane in enveloped viruses, in contrast to only encapsulation of nucleic acids by capsid proteins (27). In addition, the nature, types of organics (humic acid vs fulvic acid), and their relative abundance are known to impact virus recovery from treated biosolids (28). The virus detection efficacy data for different methods were retrospectively analyzed to identify the impact/correlation between the key factors such as viral structure, genome and virion type, and wastewater sample type on the relative performance of detection methods. Table 2 summarizes the number of detected viruses in both influent and effluent samples, categorized based on whether they are enveloped or non-enveloped viruses and whether they contain DNA or RNA genomes.

**TABLE 2:** Viral diversity in wastewater samples using different concentration methods

Sample and virus types	No. of viruses detected by the methods					
	IMNP		EMF		PEG	
Wastewater sample	Influent	Effluent	Influent	Effluent	Influent	Effluent
Variety of enveloped viruses	5	4	3	4	3	3
Variety of non-enveloped virus	16	7	13	4	17	13
Average variety of enveloped viruses	3.33	3.33	2.33	3.33	2.33	3
Average variety of non-enveloped viruses	12.67	4	8.33	2	12.33	9.33
DNA virus	7	0	8	0	11	0
RNA virus	14	11	8	8	9	16
Total detected virus	21	11	16	8	20	16

#### Viral structure

The viruses detected in the influent and effluent wastewater samples were predominantly non-enveloped viruses (Table 2). Previous studies have highlighted that enveloped viruses generally exhibit more rapid decay rates in untreated and pasteurized wastewater than non-enveloped viruses (29–31). This observation corroborates the findings of earlier research, which demonstrated that non-enveloped viruses are more resistant to various environmental conditions, including temperature, pH, and disinfection, compared with enveloped viruses. This is primarily attributed to the susceptibility of the lipid layer of enveloped viruses, which are more sensitive to environmental conditions (27, 32).

Based on the results obtained, it is evident that in the influent sample, the average number of enveloped viruses detected in IMNP samples (*n* = 3.33) was greater than in PEG and EMF samples (*n* = 2.33), suggesting better efficiency. The average number of detected enveloped viruses in all effluent samples is comparable with influent; however, the average number of detected viruses in the IMNP and EMF methods is slightly greater than that in the PEG method. Greater average numbers of non-enveloped viruses were detected by PEG (*n* = 12.33) and IMNP (*n* = 12.67) than in EMF (*n* = 8.33) method. There may be other key factors that play a more significant role in the detection of non-enveloped viruses. A decrease in the number of detectable non-envelop viruses was noted across the wastewater treatment train from influent to effluent. Regardless, the PEG method extracted more types of non-enveloped viruses (*n* = 13), nearly twice as many as IMNP and approximately three times more than EMF. Previous studies reported that different viral concentration methods showed different recovery efficiencies based on the type of viruses, distinguishing between enveloped and non-enveloped (7). In contrast to the expected trends, no consistent pattern for either enveloped or non-enveloped viruses was observed across the methods or sample types. Additional factors must be considered when selecting an appropriate viral concentration technique for studying viruses. Primarily, it is essential to identify the target virus and consider its structure when designing experiments for WBE studies to mitigate the risk of obtaining false negative results.

#### Nucleic acid structure

The data on viruses detected in the influent and effluent wastewater samples were retrospectively analyzed to find a correlation between the nucleic acid structure and the efficiency of the viral concentration method. During this study, both RNA and DNA viruses were detected in the influent and effluent samples, including 13 DNA and 22 RNA viruses (Table 1). Some of the most prevalent viruses in influent samples with various nucleic acid compositions are Astrovirus (ssRNA) viruses, Rotavirus A and C (dsRNA), Torque Teno virus, and Human polyomavirus 6 (ssDNA), and JC polyomavirus (dsDNA) viruses. The diversity of genomic and capsid structures in detected viruses processed by various concentration methods differs (Table 2). In influent samples, the PEG method exhibited greater efficiency in detecting DNA viruses, identifying 11 compared with the eight and seven DNA viruses detected by EMF and IMNP, respectively. This is in line with a previous report, indicating that the PEG method demonstrated less efficiency in the recovery of RNA viruses in raw wastewater samples using the RT-PCR detection technique compared with the phase separation method (33). The IMNP method extracted a significant number of RNA viruses (*n* = 14), which shows the efficiency of IMNP in detecting RNA viruses compared with PEG and EMF, respectively. In other words, the selection of the viral concentration method is affected by the nucleic acid structure of the target virus.

In effluent samples, all the detected viruses were RNA viruses, with no DNA viruses observed. Based on the results, PEG method demonstrated a higher efficacy in isolating RNA viruses than IMNP and EMF methods. A comparative analysis of the abundance of detected DNA and RNA viruses in influent and effluent samples revealed a significant difference in the detectability of viruses based on their genome. Although the abundance of DNA viruses in influent samples was significant, none were detected in effluent. This observation may suggest that RNA viruses exhibit greater resistance to UV contradicting prior studies suggesting that double-stranded viruses are generally more resistant than single-stranded viruses due to their ability to repair their genome during the replication process in the host cell (24, 34, 35).

#### Impact of wastewater treatment level on detectable viruses

**Fig 4:**
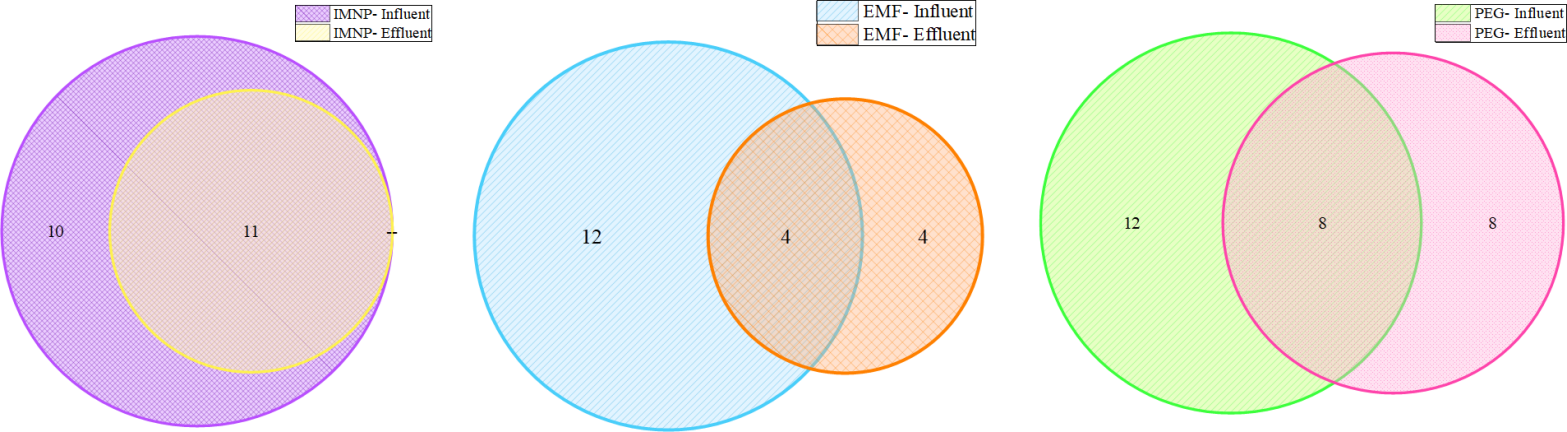
Venn diagram of viral distribution in influent and effluent samples.

The impact of wastewater treatment levels on the type of detectable virus by different viral concentration methods was analyzed, and the results are presented as a Venn diagram (Fig. 4). In the IMNP sample, all detected viruses in the effluent samples are subgroups of those detected in influent samples, suggesting that the treatment level does not influence the efficiency of IMNP method. Notably, if a virus is detected in the effluent sample processed by IMNP method, it can also be detected in the influent sample. However, the results from the EMF and PEG methods reveal that although the number of detected viruses in the influent sample is greater than in the effluent samples, some viruses not detected in the influent samples were detected in the effluent samples. Examples include Rotavirus A, Norovirus GII, Norovirus GI strain Hu/JP/2000 /GI.6[PNA1]/WUG1, and Hepatitis C virus genotype 6 all of which were detected in the effluent sample processed by the EMF method but were not detected in the corresponding influent sample. A similar trend was observed for viruses processed by the PEG method for Rotavirus A, Norovirus GII GII.NA2[PNA2], Norovirus GII.P7_GII.6, Norovirus GI strain Hu/BD/2011 /GI.7[PNA2]/Dhaka1882, Norovirus GI strainHu/JP/1998 /GI.6[PNA4]/No20-Saitama-98–17, NorovirusGI/Hu/JP/2007 /GI.P3_GI.3/Shimizu/KK2866, Aichivirus A, and Salivirus. These results are consistent with the previous studies reporting that PEG and similar viral concentration methods co-concentrate the organic molecule along with viral particles (28). These elevated levels of organic molecules (such as humic material) are known to impart PCR inhibition, which can be overcome by sample dilution (28). The fact that influent samples have a higher concentration of humic material than the effluent sample indicates that the non-detection of some viruses in the influent sample might be due to higher levels of humic substances compared with effluent samples. Immunocapture methods (such as IMNP) are known for cleaner sample concentrations because of higher rejection of organic contaminants during the concentration step, therefore minimizing PCR inhibition (34, 35). In addition, fecal residues and urine constituents are known to impart PCR inhibition, which can be reduced by immunomagnetic purification of samples (36). This can explain the results of the IMNP method in this study where the virus detected in the effluent sample was also detected in the influent sample.

**Fig 5:**
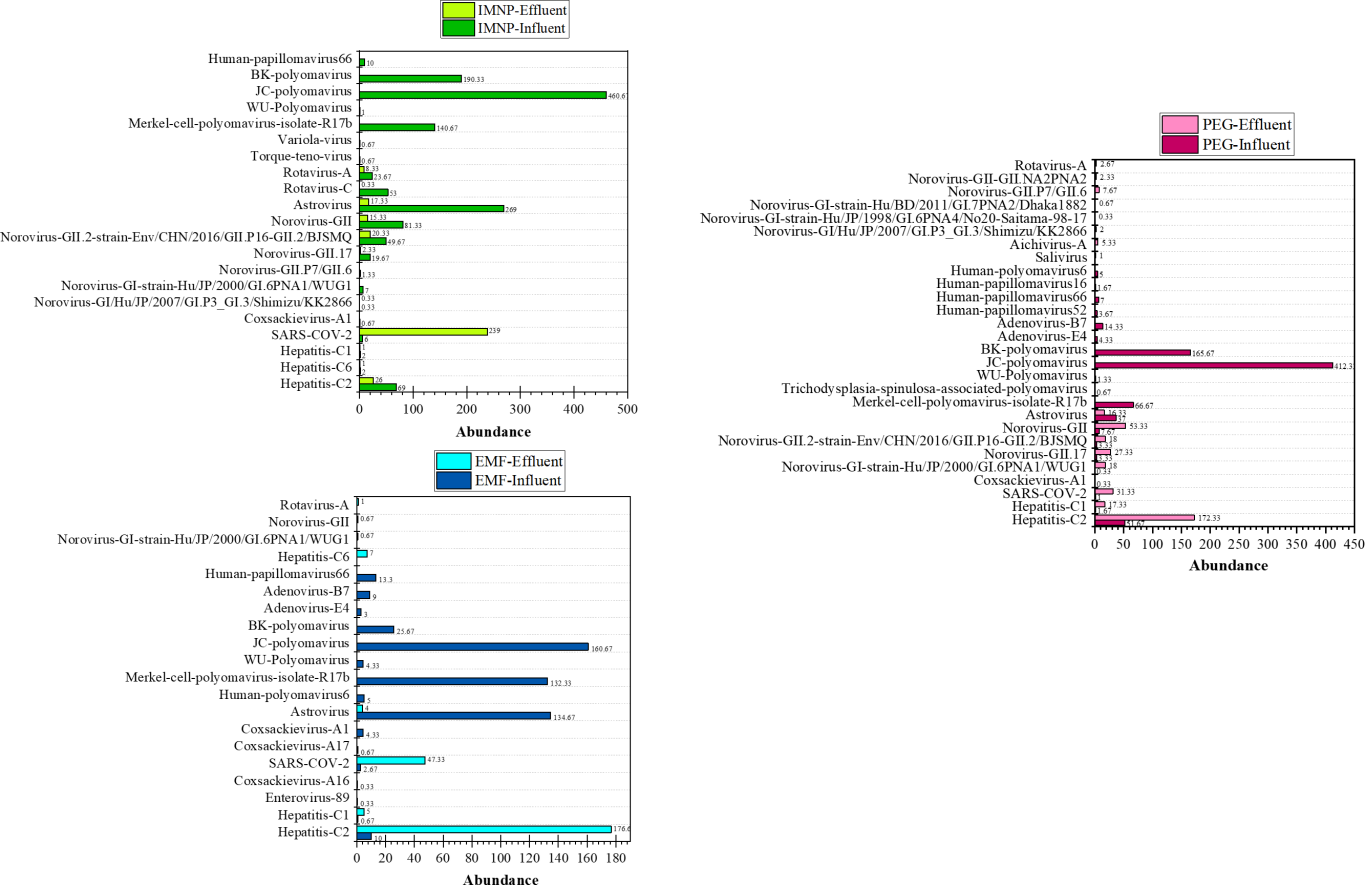
Absolute abundance of detected viruses in influent and effluent samples processed by the three concentration methods.

The abundance of detected viruses in both influent and effluent samples is presented in Fig. 5. The results indicate that the abundance values ranged from 0.33 to 460.67 reads in influent samples and from 0.33 to 239 reads in effluent samples. The results show that JC Polyomavirus was the most abundant virus identified in influent sample by all the IMNP, EMF, and PEG methods. Observations highlight that detected viruses in influent and effluent wastewater samples concentrated by different techniques show varying abundances, indicating that the concentration method impacts not only the type of detected viruses but also the levels of abundance. In influent samples, the abundance of detected viruses does not exhibit a consistent trend, making it challenging to compare the efficiency of concentration methods based on virus abundance.

According to the results, it is evident that certain viruses were exclusively detected in influent wastewater samples. The absence or reduction in their abundance in effluent samples suggests that the wastewater treatment process has caused these viruses to become inactive or decreased their levels (26). These findings align with prior studies that noted a higher abundance of bacteriophages in untreated samples (26). However, some viruses were undetected in influent samples but were identified in corresponding effluent samples. The EMF concentration method, for instance, failed to detect specific viruses, such as Rotavirus A, in the influent sample but successfully identified them in the effluent sample. A similar scenario was observed for Aichivirus-A and Salivirus in samples processed by the PEG method. Previous research has indicated that treatment processes effectively remove bacteria and protozoa but are not entirely efficient in eliminating all human viruses detected in effluent samples, including adenoviruses and enteroviruses (8). The higher PCR inhibitor concertation in influent samples might be a reason for the non-detection of some viruses in influent. The lower concentration of PCR inhibitors in effluent may have allowed us to overcome the PCR inhibition barriers, resulting in detecting some viruses only in treated samples (28).

A comparison of the absolute abundance of detected viruses in influent and effluent samples processed by the same concentration method revealed an increase in the absolute abundance of certain viruses in the effluent. Specifically, in the IMNP method, SARS-COV-2 exhibited higher abundance; in the EMF method, Hepatitis C virus genotype 2, Hepatitis C virus genotype 6, and SARS-COV-2 were higher; and in the PEG method, Norovirus GII, Norovirus GII GII.NA2[PNA2], Norovirus GII.17, Norovirus GII.2 strain Env/CHN/2016/GII.P16-GII.2/BJSMQ, Hepatitis C virus genotype 2, Hepatitis C virus genotype 6, SARS-COV-2, and Norovirus GI strain Hu/JP/2000 /GI.6[PNA1]/WUG1 showed increased abundance compared with the corresponding values in influent samples. In previous research conducted by Corpuz et al., it was reported that SARS-COV-2 was detected in effluent samples but not in influent. The absence of the virus in influent and its presence in effluent suggest that embedded viruses were released, possibly due to protection during the treatment process. Studies have shown that some viruses tend to adsorb on non-organic matter and can be protected by suspended solids, influencing the final concentration of the virus (27, 37, 38). The higher abundance of these viruses in effluent samples may be attributed to the treatment process’s efficacy in reducing other microbial and organic contaminants, allowing their detection. This could explain the detection of some viruses in effluent samples that were not identified in influent samples (26, 28). Based on the diversity in the variety and abundance of detected viruses in effluent samples, various viral concentration methods did not exhibit equal effectiveness at the treatment level. The treatment process’s efficacy is determined by quantifying and identifying viruses in effluent samples (39). As Rock et al. discussed, it is critical to consider the degree of inhibition present in wastewater samples prior to the detection method, which is consistent with our results. Therefore, identifying the type, assessing the level, and removing inhibitors in samples can help minimize the chances of false-negative results from the detection analysis (28).

### Conclusion

WBE is widely used to provide valuable information about public health and diseases in a community. Nevertheless, the presence of bias in viral detection methods highlights the critical need for meticulous experimental design and the selection of appropriate viral concentration techniques. The potential occurrence of false negative results poses a notable risk to the credibility of WBE applications and necessitates designing concentration methods based on the target virus. The viral structure, including the lipid layer’s presence or absence, is not the only determining factor in designing the most efficient concentration techniques. The ultimate effects of microbial quality of wastewater discharge in source waters and reuse applications have a direct impact on the potential risk of exposure to the end users, highlighting the importance of robust detection methodologies for avoiding potential false negative results. The observed differences in viral variety and abundance trends between influent and effluent wastewater samples processed using various concentration methods require further investigation to identify the types and degrees of different inhibitors across detection processes. Consequently, additional research is recommended to identify optimal viral surrogates based on the efficient key factors and concentration methods, thereby enhancing the reliability of detected viruses through wastewater.

## Data Availability

The next-generation sequencing data are available in NCBI under BioProject PRJNA1185467.
